# Zn-Ion Hybrid Capacitor Cyclic Stability Enhancement via Zinc Nitride Coating

**DOI:** 10.1021/acs.energyfuels.6c01083

**Published:** 2026-06-26

**Authors:** Subrata Ghosh, Giacomo Pagani, Stefanos Chaitoglou, Debashis Tripathy, Andrea Lucotti, Matteo Tommasini, Carlo S. Casari

**Affiliations:** † Micro and Nanostructured Materials Laboratory — NanoLab, Department of Energy, 18981 Politecnico di Milano, via Lambruschini, 8, 20156 Milano, Italy; ‡ Department of Physics, Applied Science Cluster, School of Advanced Engineering, UPES, Dehradun 248007, India; § Department of Applied Physics, University of Barcelona, C/Martí i Franques, 1, 08028 Barcelona, Catalunya, Spain; ∥ ENPHOCAMAT Group, Institute of Nanoscience and Nanotechnology (IN2UB), University of Barcelona, C/Martí i Franques, 1, 08028 Barcelona, Catalunya, Spain; ⊥ Department of Chemistry, 2152 University of Cambridge, Cambridge CB2 1EW, U.K.; # Department of Chemistry, Materials and Chemical Engineering “Giulio Natta”, 18981 Politecnico di Milano, Piazza Leonardo da Vinci 32, 20133 Milano, Italy

## Abstract

By depositing zinc nitride on bare Zn, the present study showcases that nitride coating suppresses formation of the insulating oxide/hydroxide layer, controls the growth of Zn nucleation, and forms a uniform network of Zn nanostructures. Hence, an aqueous zinc-ion hybrid microcapacitor delivers better charge-storage performance and long lifespan with capacitance retention of 110% after 10 000 cycles.

## Graphical abstract

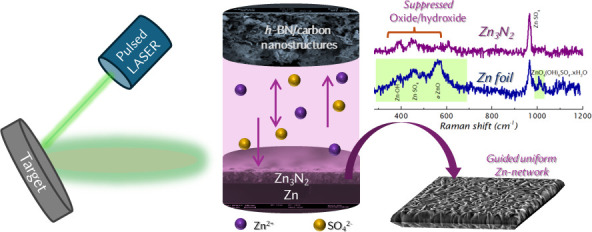

Rapidly expanding demand for renewable energy is intensifying the development of suitable electrochemical energy storage devices such as batteries, supercapacitors, metal-ion capacitors, and fuel cells. Metal-ion hybrid electrochemical capacitors hold promise, as they bridge the gap between high-energy-density batteries and high-power-density supercapacitors.^1–3^ While significant research progress on Li-ion and Na-ion hybrid capacitors is evidenced, Zn-ion hybrid electrochemical capacitors (ZICs) show appealing features, including volumetric (gravimetric) capacity of 5855 mAh/cm^3^ (820 mAh/g), low redox potential of −0.76 V against standard hydrogen electrode, low polarizability compared to Al and Mg, abundance, greater safety than Li, Na, Mg, and K, and low cost.^4,5^

In most aqueous ZIC configurations, while engineering the carbon-based cathode materials has received significant attention, the anode material most often used is a bare Zn foil. The main limitations to obtaining high-performance Zn-ion storage devices are stripping/plating of Zn/Zn^2+^, formation of an insulating oxide and hydroxide layer, and unstable dendrites due to an uneven electric field and nonuniform ion distribution. To overcome these issues, besides the cathode material engineering, Zn foil modification, design of a porous Zn structure, coating a carbon-based layer on the Zn surface, synthesizing Zn-based nanocomposites, and separator modification have been proposed so far.^6–8^ In a early report, the micro-ZIC with an oxidized carbon nanotube (CNT) cathode and Zn anode delivers the maximum areal capacitance of 20 mF/cm^2^.^4^ The aqueous device featuring laser-assisted turbostatic graphene with ZnAc_2_ exhibited a capacitance of 5.8 mF/cm^2^ at a current density of 0.2 mA/cm^2^.^9^ A CNT-based ZIC with KOH–H_2_SO_4_ dual-pH electrolytes exhibited a capacitance of 18.7 mF/cm^2^ at 5 mV/s.^10^ Nevertheless, the areal capacitance of thin-film micro-ZICs is low, and hence, there is a need for engineering a Zn anode and a suitable cathode material.

Here, a compact coating of nitride with a thickness of around 160 nm on the 250 μm Zn foil was synthesized by pulsed laser deposition (PLD) under N_2_ gas (see cross-sectional image in Figure S1) and annealed at a temperature of 300 °C (below the melting point of zinc: 420 °C) to obtain better structural quality. The sample named hereafter is compact_Zn. The Raman spectrum and surface texture of zinc nitride-coated Zn foil are shown in Figure 1a,b. The Raman spectrum of the compact_Zn film consists of two broad regions: 100–300 cm^–1^ and 300–800 cm^–1^. In the frequency range of 100–300 cm^–1^, the peak is deconvoluted into a peak at 238 cm^–1^ and another at 267 cm^–1^. The peak at 267 cm^–1^, which was also observed in a ZnO:N film but has not been seen in ZnO film, is attributed to the substitution of nitrogen for the oxygen site (N_O_).^11^ The peak at 238 cm^–1^ is ascribed to the ZnO formation and could be due to the native oxide on the surface. By deconvoluting the band in the frequency range of 300–800 cm^–1^, we found an intense peak at 573 cm^–1^ with a full width at half-maximum of 46 cm^–1^ along with shoulder peaks at around 447, 533, and 629 cm^–1^ (fitting detail is provided in Table S1). In previous reports of N-doped ZnO,^12,13^ the Raman spectrum consists of a high-intensity peak at 570 cm^–1^ and shoulder weak peaks at 280, 510, and 642 cm^–1^. In the report of a Zn_3_N_2_ film grown at 498 K using radiofrequency magnetron sputtering,^14^ the Raman spectrum shows only two sharp peaks at 257 and 565 cm^–1^. Moreover, the intensity of these two Raman peaks in N-doped ZnO is proportional to the N concentration.^12^

**Figure 1:**
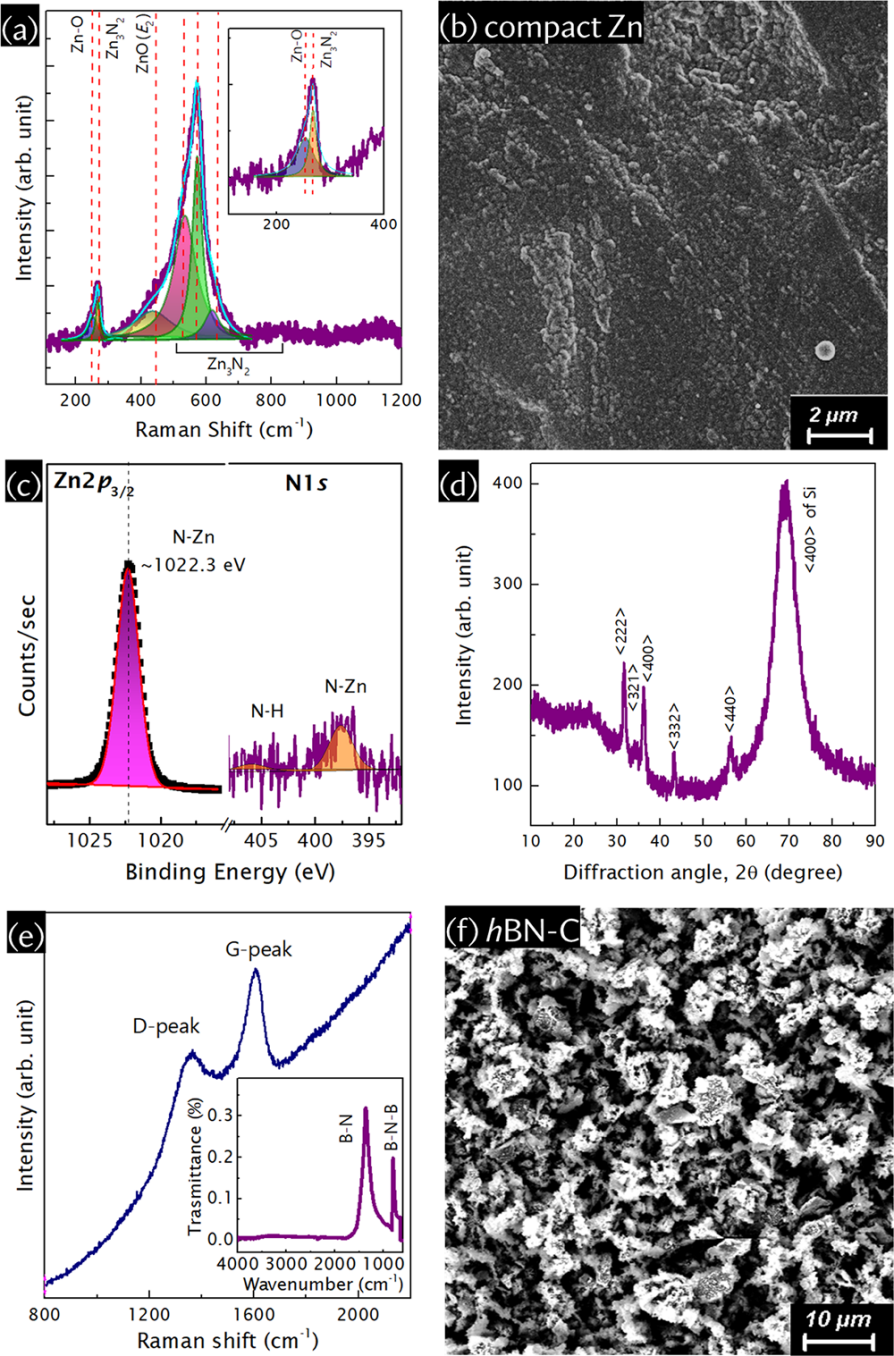
(a) Raman spectra, (b) electron micrograph, (c) high-resolution Zn 2*p*_3/2_ and N 1*s* X-ray photoelectron spectra, and (d) X-ray diffraction pattern of compact_Zn film. (e) Raman spectrum and (inset) infrared spectrum and (f) scanning electron micrograph of *h*BN-C nanostructure.

The broadened peaks at 238 and 573 cm^–1^, accompanied by shoulder peaks in our case, could be due to the poor crystalline quality. Similar broadened Raman peaks of Zn_3_N_2_ have also been reported^15^ and were attributed to the conversion of Zn_3_N_2_ to ZnO upon exposure to an ambient environment. The obtained Raman spectra of our compact film could be related with the presence of ZnO since the peak at around 571 cm^–1^ can be assigned as the A_1_ longitudinal optical mode^16^ and the sharp peak at around 440 cm^–1^ as the E_2_^high^ mode).^17^ However, we have not noticed a peak as sharp as seen in ZnO (437 cm^–1^).^18,19^ We do agree that the physisorption of oxygen on the surface is unavoidable, which resulted in the appearance of peaks at 238 and 447 cm^–1^. However, the peaks with frequencies of 267, 533, 573, 628, and 847 cm^–1^ do not belong to first- or second-order Raman modes of ZnO.^12^ Since the Zn target was ablated in PLD under the N_2_ environment at 10^–2^ Pa without the use of oxygen during the deposition, the Raman spectroscopic analysis confirmed that the compact coating is zinc nitride.^14,15^

The zinc nitride formation was further confirmed by X-ray photoelectron spectroscopy (XPS) and X-ray diffraction (XRD) results. The obtained peak at around 1022 eV in the high-resolution Zn 2*p*_3/2_ spectrum (Figure 1c left panel and Figure S2) and the large chemical shift in the N 1*s* spectrum from the free amine group (Figure 1c) of compact_Zn certainly confirms the presence of Zn_3_N_2_.^20^ Figure 1d reveals diffraction peaks centered at 31.6, 34.2, 36.1, 43.2 and 56.5°, corresponding to diffractions from the ⟨222⟩, ⟨321⟩, ⟨400⟩, ⟨332⟩, and ⟨440⟩ planes of Zn_3_N_2_.^20^ The peak centered at 69.2° corresponds to the ⟨400⟩ Si substrate diffraction.

The anode material for the ZIC fabrication in our work is hexagonal boron nitride-coated carbon (*h*BN-C) nanostructure, which was prepared by a one-step PLD method^21^ and vacuum-annealed at 900 °C for 1 h. The thickness of *h*BN-C grown on the Si substrate is estimated to be 22.9 ± 4.4 μm. The Raman spectrum of *h*BN-C nanostructures is characterized by the presence of the disordered D band and graphitic G band (Figure 1e). We could not identify the peak related to the *h*-BN nanostructure, as its position overlapped with the spectral region of amorphous carbon. The presence of *h*BN in the structure was confirmed from the infrared spectrum (inset of Figure 1e), which contains sharp peaks at around 804 and 1367 cm^–1^ representing the *out-of-plane* bending mode and *in-plane* stretching mode of *h*-BN, respectively.^22^ The morphology of the porous *h*BN-C nanostructure is shown in Figure 1f. The elemental mapping of the EDX image (Figure S3) confirms the uniform and homogeneous distribution of each element.

Figure 2a shows the cyclic voltammogram of the *h*BN-C//compact_Zn thin-film device at various scan rates ranging from 20 mV/s to 2 V/s. Almost unchanged voltammograms even at high scan rates ensure the high-power capability of the device (Figure 2a). To confirm the better performance of the nitride coating, the cyclic voltammograms of hybrid capacitor devices, namely, *h*BN-C//bare_Zn, and *h*BN-C//ablated_Zn, are provided in Figure S4. The ablated_Zn was prepared by ablating Zn using the same laser of PLD and mounting the Zn foil in the target position. The morphology and Raman spectra of bare_Zn and ablated_Zn are also provided in Figure S5. The obtained areal capacitance (capacity) of *h*BN-C//compact_Zn is 53.4 mF/cm^2^ (15.31 mAh/cm^2^) at 20 mV/s, which is higher than that of *h*BN-C//ablated_Zn (21.4 mF/cm^2^, 6.2 mAh/cm^2^) and *h*BN-C//bare_Zn (34.3 mF/cm^2^, 9.95 mAh/cm^2^). At a high scan rate of 2 V/s, the areal capacitance (capacity) of *h*BN-C//compact_Zn is estimated to be 13.82 mF/cm^2^ (3.96 mAh/cm^2^). The electrochemical kinetics of the *h*BN-C//compact_Zn device was probed from the relationship between current (*i*) and scan rate (*v*) by the equation *i* = *av* ^*b*^, where *a* and *b* represent dynamic constants. An obtained *b* value of 0.74 indicates that the process is a combination of surface-driven capacitive and diffusive processes (Figure 2c). After the estimation of those contributions (Figure S6), we found that the capacitive contribution becomes dominant at a higher scan rate (Figure 2d). The cycle stability study also revealed that the nitride coating is beneficial to achieve an ultralong cycle stability (110% after 10 000 cycles) compared to the ZIC devices with ablated_Zn (101%) and bare_Zn (84%) (Figure 2e). The higher cycle stability of the ablated_Zn device compared to that of bare_Zn device could be attributed to the change in texture of Zn. Since the laser ablation does not alter the crystal structure of Zn, it indeed changes the surface morphology in terms of texture and roughness (Figure S3c). This change in texture provides additional adsorption sites for zinc ions and hence a better electrode–electrolyte interface and higher electrochemical stability. The ultralong cycle stability of *h*BN-C//compact_Zn could be due to the electrochemical-activation-associated changes in both the *h*BN-C cathode and compact_Zn during charge–discharge, as seen from post-mortem analysis.^23^ From the electrochemical impedance spectra, a lower equivalent series resistance is obtained for *h*BN-C//compact_Zn (1.14 Ω) compared to *h*BN-C//bare_Zn (1.58 Ω) and *h*BN-C//ablated_Zn (1.28 Ω) (Figure S7). Moreover, the compact_Zn device also offers better electrolyte ion diffusion, as it shows a lower Warburg coefficient (σ) of 430.19 Ω/s^0.5^ (Figure 2f) compared to *h*BN-C//bare_Zn (647.36 Ω/s^0.5^) and *h*BN-C//ablated_Zn (660.75 Ω/s^0.5^). This fact ensures that the nitride coating promotes the kinetics of electrolyte ion transportation,^24^ and we anticipate further enhancement in performance by improving the nitride coating quality through deposition process parameter control. The Zn-ion storage performance of our proposed micro-ZIC is compared with the existing literature in Table S2.

**Figure 2:**
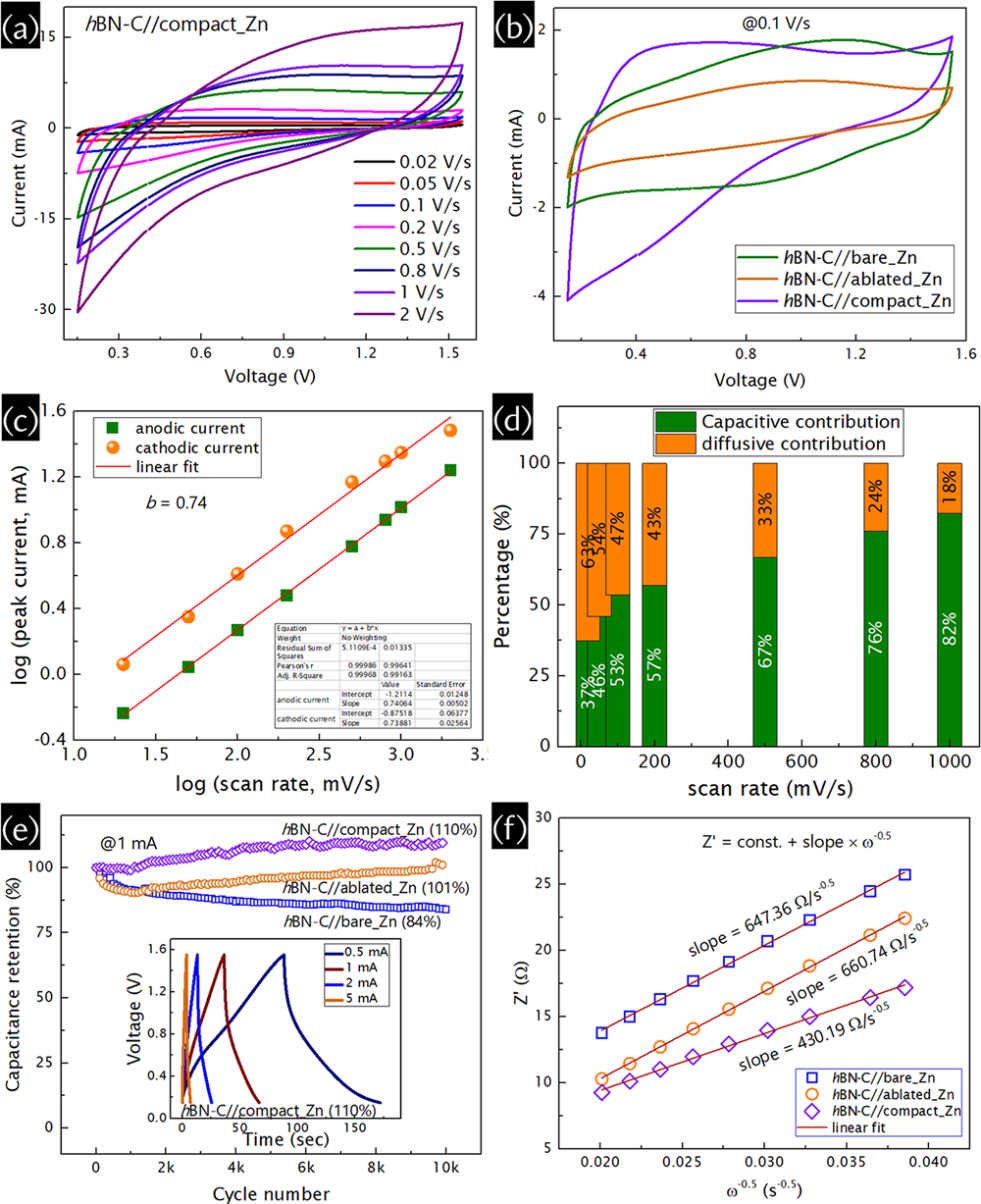
(a) Cyclic voltammogram of the *h*BN-C//compact_Zn device at various scan rates. (b) Comparative cyclic voltammograms of all devices. (c) Logarithmic plot of cathodic and anodic peak currents versus scan rates and (d) comparison of contributions of the *h*BN-C//compact_Zn device at different scan rates. (e) Cycle test and (f) *Z*′ vs ω^–0.5^ plot for all studied microdevices. The aqueous electrolyte was 2 M ZnSO_4_. The inset of (e) shows the charge–discharge profiles of *h*BN-C//compact_Zn at various currents.

We also performed post-mortem analysis to probe the ZIC performance further. Analysis of the Zn surface morphology after 10 000 charge/discharge cycles suggests that bare_Zn suffers from a severe corrosion reaction, resulting in uneven and chaotic deposition of Zn structure on Zn foil (Figure 3a). Relatively similar uneven Zn deposition, including tip growth, is also noticed for ablated_Zn (Figure 3b) after the electrochemical cycles. For compact_Zn, the nitride coating guided the Zn nucleation uniformly to form an interconnected Zn network (Figure 3c), which offers electrolyte ions effective access to the Zn surface and prevents the localized ion accumulation.^25^ Raman spectra of bare Zn foil and modified Zn were recorded after the electrochemical investigation (Figure 3d) to gain further insights on the surface species. Raman spectra of both bare_Zn and ablated_Zn show a peak at around 565 cm^–1^, which represents the amorphous ZnO formation,^26^ whereas it is absent for the compact_Zn. Second, one can hardly observe any signature of ZnO_4_(OH)_6_SO_4_·*x*H_2_O in ablated_Zn and compact_Zn compared to the bare_Zn counterpart, indicating the effectiveness of surface treatment in preventing undesired reactions. Similar observation is also confirmed from the post-mortem XRD result (Figure 3e) of compact_Zn, where diffraction peaks centered at 36.3, 39, 43.2, 54.4 and 70° correspond to diffractions from the ⟨101⟩ planes of ZnO and ⟨101⟩, ⟨102⟩, ⟨103⟩, and ⟨104⟩ planes of metallic Zn. Meanwhile, diffraction peaks from the zinc sulfate hydroxide hydrates are not observed.^6^ The presence of sulfur-containing element/compound is confirmed from both Raman and XPS spectra of all Zn anodes (Figures 3d and S7). However, zinc nitride can transform into its oxide counterpart in a top-down manner, as the surface of the nitride interacts with the aqueous electrolyte during the electrochemical process. Hence, O–Zn bonds are evidenced in the high-resolution Zn 2*p*_3/2_ XPS spectra (left panel of Figure 3f) but no contribution of Zn­(OH)_2_ formation (binding energy ≈ 1022.2 ± 0.5 eV^27^) in compact_Zn after the electrochemical charge–discharge. However, deconvolution of the O 1*s* spectrum shows a little contribution of Zn­(OH)_2_, which is in good agreement with the Raman spectroscopic result. It is worth mentioning that the presence of high-resolution N 1*s* spectrum (right panel of Figure 3f), as obtained in as-grown compact_Zn (right panel of Figure 1c), reveals the stability of nitride characteristics even after prolonged cycling. These observations reveal that the incorporation of nitrogen and replacing native surface oxygen is highly beneficial to improve the Zn metal stability and hence the Zn-ion storage performance. Chronoamperometry and investigation of a symmetric cell of zinc nitride may provide more insights, which could be a subject of further research.^28,29^

**Figure 3:**
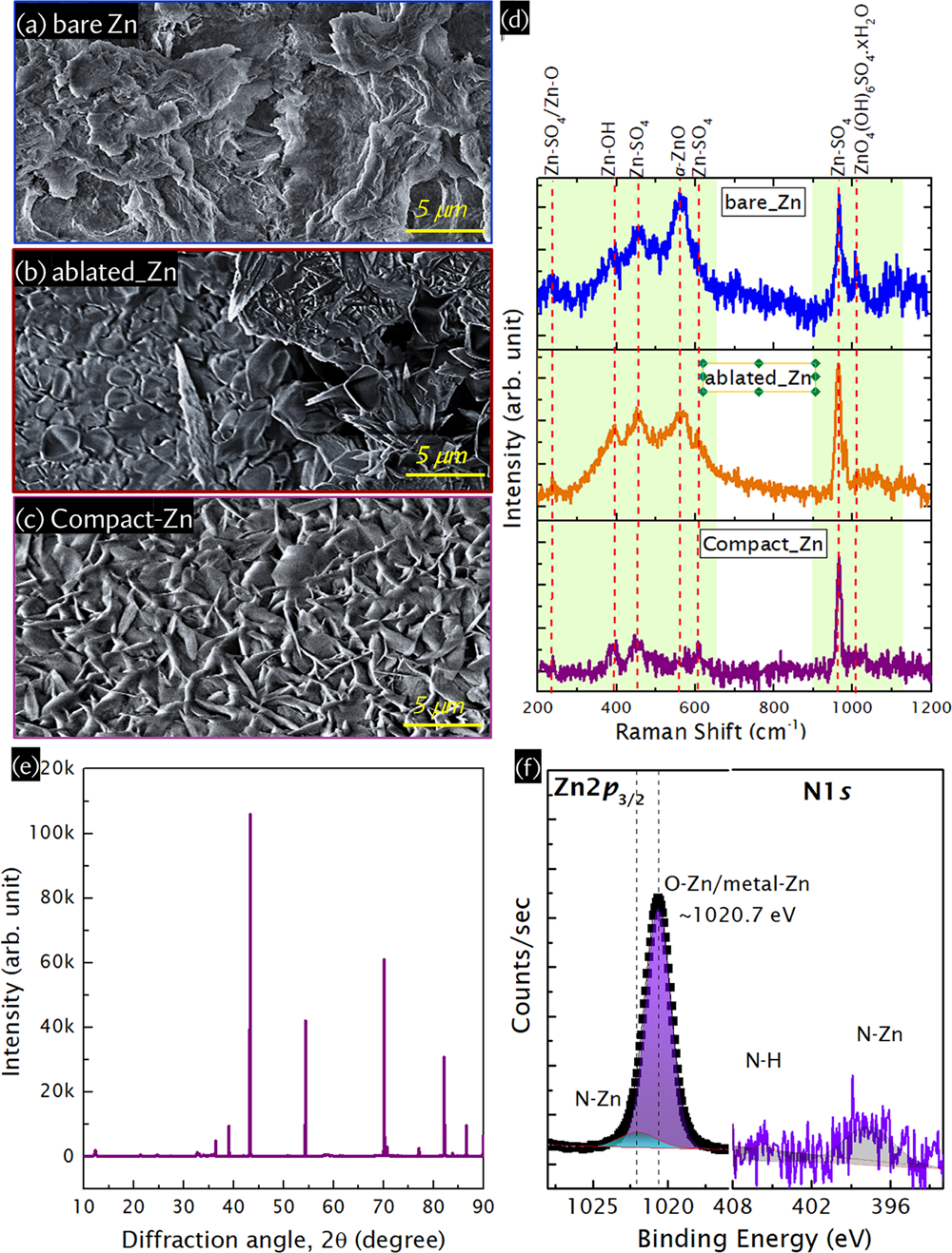
Post-mortem results. (a–c) Scanning electron micrographs of (a) bare_Zn, (b) ablated_Zn, and (c) compact_Zn after electrochemical investigations. (d) Raman spectra of bare_Zn, ablated_Zn, and compact_Zn after the electrochemical test. (e) X-ray diffraction pattern and (e) high-resolution Zn 2*p*_3/2_ and N 1*s* X-ray photoelectron spectra of compact_Zn.

In summary, we have proposed a strategy of zinc nitride coating to enhance the charge storage performance of ZICs. The nitride coating not only suppressed formation of the insulating oxide/hydroxide layer but also offered control of zinc deposition by forming a uniform interconnected Zn nanostructure, which resulted in better electrolyte ion diffusion and excellent electrochemical stability over prolonged charge–discharge cycles. The present finding underscores the importance of the nitride coating and provides a new way to develop sustainable and safe aqueous zinc-ion hybrid electrochemical capacitors.

## Supplementary Material



## Data Availability

All data from this study are available in the article and its Supporting Information.

## Notes

Experimental details, additional electron micrograph, EDX results, additional XPS results, Raman spectrum and fitting details, additional electrochemical results and capacitive contribution calculation details, and comparison table of thin-film-based Zn-ion hybrid electrochemical capacitor (PDF)
