# Supplementary material for: Zn-Ion Hybrid Capacitor Cyclic Stability Enhancement via Zinc Nitride Coating
Source: Energy Fuels. 2026 Jun 26;40(27):14918–22. doi: 10.1021/acs.energyfuels.6c01083 (PMC13359099; doi:10.1021/acs.energyfuels.6c01083)
Supplement: Supplementary file 1 [file ef6c01083_si_001.pdf]

SUPPORTING INFORMATION

Zn-ion hybrid capacitor cyclic stability enhancement via Zn-nitride coating

Subrata Ghosh[1,2]*, Giacomo Pagani[1], Stefanos Chaitoglou[3,4], Debashis Tripathy[5], Andrea Lucotti[6], Matteo Tommasini[6], Carlo S. Casari[1]*

[1] *Micro and Nanostructured Materials Laboratory — NanoLab, Department of Energy, Politecnico di Milano, via Ponzio 34/3, Milano, 20133, Italy*
[2] *Department of Physics, Applied Science Cluster, School of Advanced Engineering, UPES, Dehradun, 248007, India*
[3] *Department of Applied Physics, University of Barcelona, C/Martí i Franques, 1, 08028, Barcelona, Catalunya, Spain*
[4] *ENPHOCAMAT Group, Institute of Nanoscience and Nanotechnology (IN2UB), University of Barcelona, C/ Martí i Franques, 1, 08028, Barcelona, Catalunya, Spain*
[5] *Department of Chemistry, University of Cambridge, Cambridge CB2 1EW, UK*
[6] *Department of Chemistry, Materials and Chemical Engineering "Giulio Natta", Politecnico di Milano, Piazza Leonardo da Vinci 32, 20133 Milano, Italy*

Corresponding author email: subrata.ghoshk@rediffmail.com or subrata.ghosh@upes.ac.in (S.G.) and carlo.casari@polimi.it (C.S.C.)

1. Experimental Methods

1.1. Synthesis of electrode material

Anode preparation: Bare Zn foil is used as received, without any modification. Zn compact film was deposited on Zn foil using a Nd: YAG pulsed *ns*-laser (2nd harmonic at 532 nm, pulse duration 5-7 ns, repetition rate 10 Hz) in a pulsed laser deposition system. The target used for this deposition is 1-inch diameter Zn foil. In this case, the rotation and the vertical movement of the target allowed the whole Zn target to be ablated during the deposition. The deposition pressure maintained was 2 Pa for 2 min deposition time under high pure N2 gas environment, and the laser fluence was 6.5 J/cm^2 (corresponding laser power of about 410 mJ). The distance between target and substrate was 4 cm. Before deposition, the chamber was evacuated down to 10^{-3} Pa using a scroll pump and a turbomolecular pump. After the deposition, the target Zn was also used as an anode and termed as ablated_Zn. The compact film formed on the Zn foil was taken out from the chamber after venting, and vacuum annealed at 300 °C for 1 hr in the furnace. Before this annealing step, the furnace was pumped down using a scroll pump and a turbomolecular pump. The final film obtained after annealing is termed compact_Zn.

Cathode preparation: To synthesise *h*-BN/carbon nanoflake composites, a one-step pulsed laser deposition method mentioned in our previous publication.[20] Briefly, a 1-inch BN target (purchased from Testbourne B. V.) was placed on a 2-inch carbon target (purchased from Testbourne B. V., and the purity was 99.99%) and ablated simultaneously. The substrate used was carbon paper. Same deposition parameters as mentioned before in anode preparation were employed, except for the deposition time and deposition pressures. A 2 Pa background pressure was maintained for 2 min to grow a compact film acting as a buffer layer in the first step, followed by 30 min deposition at 250 Pa to obtain a sufficiently porous material. After the deposition, the sample was annealed in a vacuum chamber at 900 °C for 1hr in the furnace.

1.2. Characterisation

The morphology of h-BN/carbon nanostructures and Zn anodes was examined by a field-emission scanning electron microscope (FE-SEM, ZEISS SUPRA 40, Jena, Germany), where an *in-lens* detector operates in a high vacuum to capture secondary electrons. Energy dispersive X-ray spectra (EDXS) were employed at the acceleration voltage of 20 kV, using AZtec software for elemental analysis. The instrument was equipped with a Peltier-cooled silicon drift detector (Oxford Instruments).

The Raman spectra of all samples were measured using a Renishaw *In via* Raman spectrometer, UK. The spectra were recorded using a 514.5 nm laser with a power of 0.4 mW on the sample, a 1800 line/mm grating spectrometer, a 50× objective lens, and 20 accumulation (10 s each). Raman spectra of Zn compact are fitted with Lorentzian lineshapes.

Micro-Fourier Transform Infrared measurements of h-BN/carbon nanoflakes deposited on silicon were performed in transmission through two co-axial Cassegrain 15× infrared objectives (one for focalising the IR beam, the other for the collection of the transmitted light) using the Nicolet Nexus interferometer coupled with a Thermo-Nicolet Continuum infrared microscope and a cooled MCT detector (77 K). The analysed area was approximately 200 µm x 200 µm.

X-ray diffraction (XRD) pattern of zinc nitride sample were recorded using the Anton Paar XRDynamic 500 multipurpose powder diffractometer in Bragg-Brentano mode with a 360 mm radius. Zinc nitride grown on Si substrate and the nitride sample after electrochemistry was exposed to Cu $K_\alpha(1+2)$ radiation with a length of 1.5418 Å, and 2θ/θ scans were conducted from 4° to 100°, with 2θ step size of 0.02°, and each measurement step lasted for 30 s.

X-ray photoelectron spectroscopy (XPS) analysis of the nitride samples were carried out with the PHI 5500 Multi-Technique System (from Physical Electronics, Chanhassen, MN, USA) using a monochromatic X-Ray source (Al K_α line of 1486.6 eV and 350 W. The area analysed had a diameter of 0.8 mm, with Survey XPS spectra having a pass energy of 187.5 eV and 0.8 eV/step, and elemental spectra having a pass energy of 11.75 eV and 0.1 eV/step.

1.3. Electrochemical test

The micro-ZICs were assembled using *h*BN-C nanocomposite as a cathode and Zn or modified Zn as an anode, a modified propylene microporous membrane (Celgard 2500, thickness ~ 25 µm, United Kingdom) as a separator, and 2M $ZnSO_4$ as an aqueous electrolyte. The thin film micro-ZIC is fabricated by sandwiching the electrolyte separator between the cathode and anode. The diameter of electrodes is 1 cm. The thickness of zinc foil is 250 µm, the compact layer of nitride coating on Si substrate is around 160 nm, and the thickness of h-BN/C nanostructures on Si substrate is 22.9 (±4.4) µm. Before device assembly, electrodes and a modified separator were immersed in 2M $ZnSO_4$ for several hours. The electrochemical performance of ZIC devices was carried out using a Swagelok Cell (SKU: ANR-B01, Singapore).

Electrochemical performances of ZICs were measured using a PALMSENS4 electrochemical workstation. Before recording the original data, the prototype ZICs were scanned at a scan rate of 100 mV/s for 1000 cycles. The areal capacitance was estimated from a cyclic voltammogram using the equation: $C_{areal} = \int I\, dV / A.v.V$, where I is the current, v is the scan rate, A is the geometric area of the electrode, and V is the voltage of the device. The electrochemical impedance spectroscopy was carried out in the frequency range of 1 Hz to 0.1 MHz at open circuit potential with a 10 mV *a.c.* perturbation.

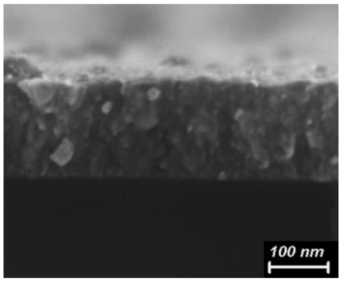

Figure S1: Cross-sectional scanning electron micrograph of Zn_compact grown on Si substrate.

Table S1: Fitting details of Raman spectrum of compact_Zn. The fitting lineshape is Lorentzian.

Position [cm^{-1}]	FWHM [cm^{-1}]	Peak Height	Peak area
237.8	55.65	262.9	22988.9
266.7	17.10	575.788	15474.3
447.49	111	218.59	38116
532.69	92.34	843.896	122403
573.25	46.13	1487.06	107743
628.54	73.81	275.674	31963.4

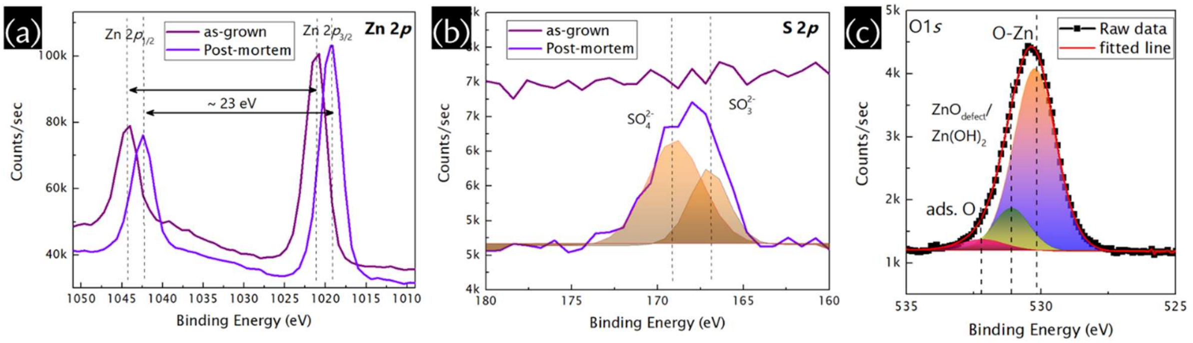

Figure S2: (a) Zn 2p and S2p X-ray photoelectron spectra of as-grown Zn_compact film and the film after electrochemical investigation (indicated as post-mortem in the Figure). (c) O1s X-ray photoelectron spectra of post-mortem Zn_compact film. For the fitting of O1*s* spectra, the preliminary fitting parameters entered from the Ref. [1] and [2]. The lineshape of the fitting is Gaussian-Lorentzian(30). The fitting detail is provided in the table below.

Position (eV)	FWHM (eV)	Area/(RSF*T*MFP)	%At Conc	Goodness of Fit
530.24	1.847	82.2669	79.22	445.377
531.1	1.6	16.4529	15.84	445.377
532.2	2	5.12063	4.93	445.377

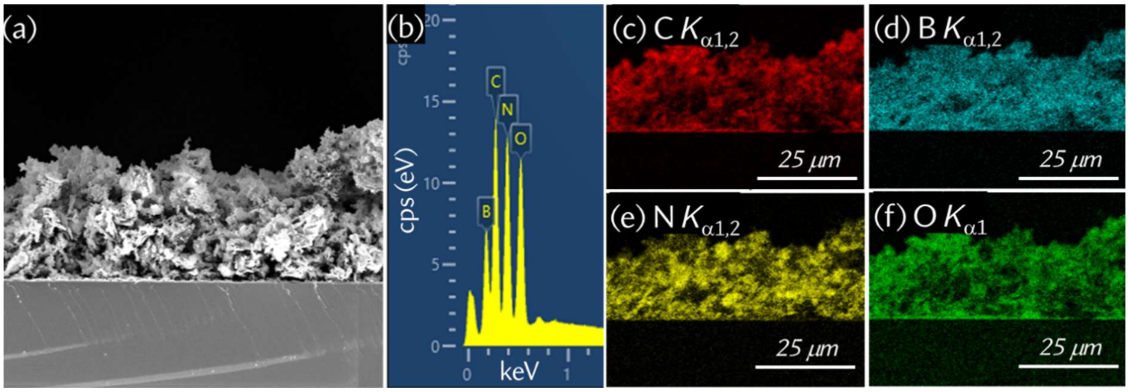

Figure S3: (a) Cross-sectional scanning electron micrograph, (b) corresponding energy dispersive X-ray spectra, and (c-f) elemental mapping of each element of *h*BN-C nanostructure.

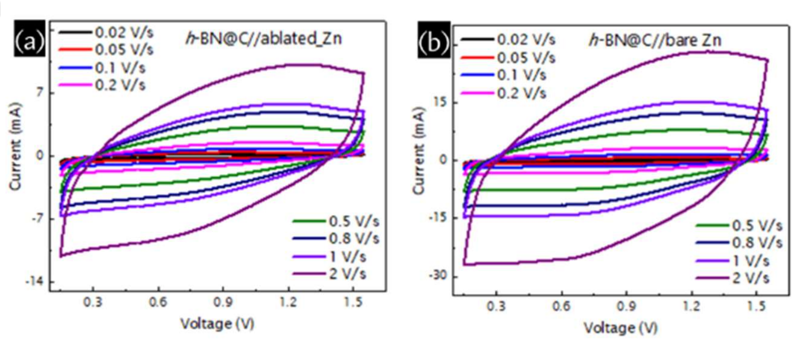

Figure S4: Cyclic voltammogram of (a) *h*BN-C//ablated_Zn and (b) *h*BN-C//bare_Zn device at different scan rates. 2M $ZnSO_4$ was used as an aqueous electrolyte

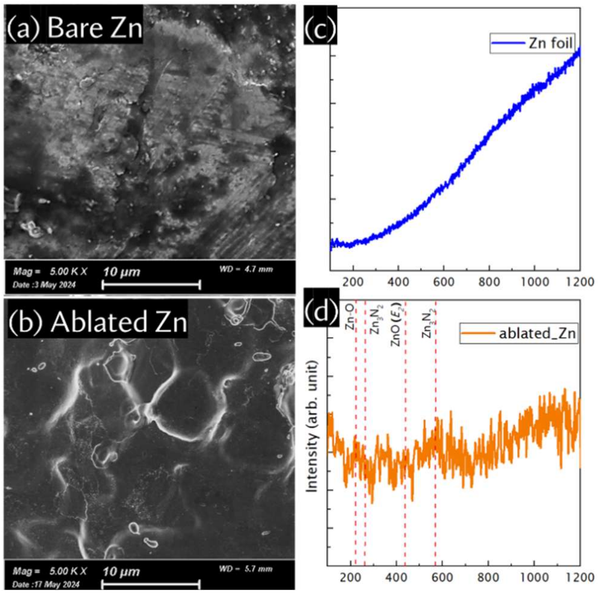

Figure S5: (a) Scanning electron micrograph and (b) Raman spectra of bare_Zn. (c) Scanning electron micrograph and (d) Raman spectra of ablated_Zn. (c) Metal does not show any Raman spectrum, and hence the Zn-foil and ablated_Zn are not exceptional (Figure 1d). The Raman spectrum of ablated_Zn spectrum appears noisier due to the irregularities on its surface resulting from laser ablation, which scatters the Raman laser and reduces the spectrum clarity.

Capacitive contribution estimation using Dunn's method

The current (*i*(*V*)) is expressed as $i(V) = k_1 v + k_2 v^{1/2}$, where, v is the scan rate, first part of the right-hand side of above expression ($k_1 v$) is capacitive-controlled and the second part ($k_2 v^{1/2}$) is diffusion controlled. For the estimation of those two contributions, we used the current (in mA) at a potential of 1.00965 V, and plotted $i(V)/v^{1/2}$ versus $v^{1/2}$ as below

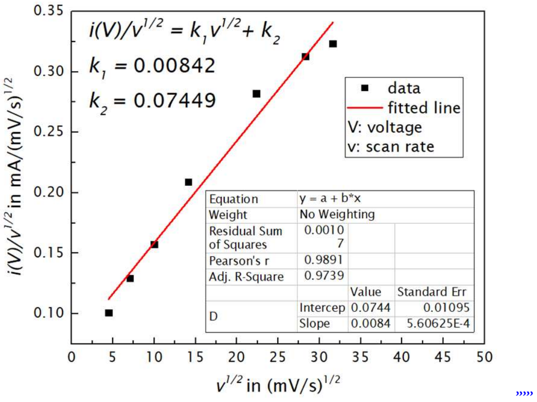

Figure *S6*: Plot of $i(V)/v^{1/2}$ versus $v^{1/2}$ for the *h*BN-C//compact_Zn device.

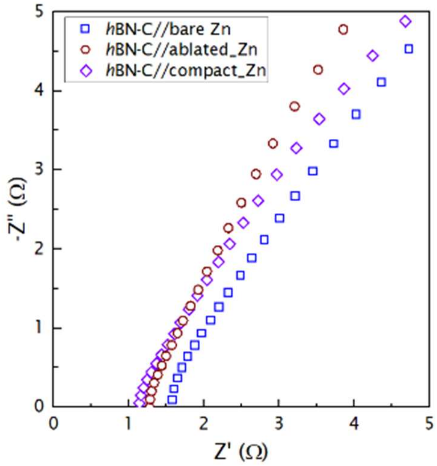

Figure S7: Nyquist plot of all studied aqueous ZIC devices. The intercept of Z'-axis at 0 is the measure of equivalent series resistance.

Note: The high-resolution Zn $2p_{3/2}$ XP-spectra can be deconvoluted with a single peak centered at 1020.71 eV, which can be ascribed as Zn-O bond formation. However, deconvoluting the spectra with two peaks provides a better fit with less residual standard deviation (RSD) compared to a one-peak fit (see the fitting below, and highlighted red box), and it also provides meaningful results, as Zn-compact is composed of a nitride component. The lineshape of the fitting is Gaussian-Lorentzian(30). The fitting details are given below in tabular form.

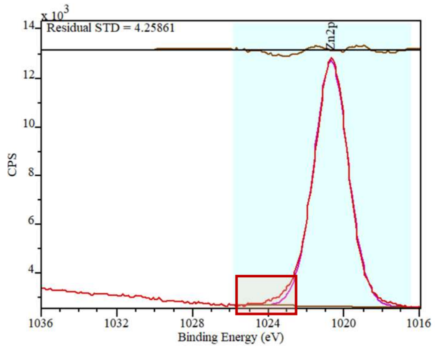

Position (eV)	FWHM (eV)	R.S.F.	Area	% Conc.	RSD
One peak fit result					
1020.71	2.082	1	868.04	100	**4.25979**
Two-peaks fit result					
1020.66	1.986	1	805.09	91.29	**2.25359**
1022.00	2.986	1	76.79	8.71	

Table S2: State-of-art thin film micro- Zn-ion electrochemical capacitor. (* represents the calculated value using the data available in the cited reference)

Cathode	Anode	Electrolyte/separator, voltage	Areal capacitance (capacity)	Cycle-life	Energy density at Power density	Ref.
hexagonal Boron nitride-Carbon nanostructure (*h*BN-C)	Compact_Zn	2M $ZnSO_4$/ modified propylene microporous membrane	53.4 mF/cm^2 (15.31 mAh/cm^2) at 20 mV/s	110% after 10k cycles	14.54 µWh/cm^2 at 0.75 mW/cm^2, 3.76 µWh/cm^2 at 19.35 mW/cm^2	This work
*h*BN-C	ablated_Zn	2M $ZnSO_4$/ modified propylene microporous membrane	21.4 mF/cm^2, 6.2 mAh/cm^2) at 20 mV/s	101% after 10k cycles	5.82 µWh/cm^2 at 0.3 mW/cm^2, 2.38 µWh/cm^2 at 12.24 mW/cm^2	
*h*BN-C	Zn foil	2M $ZnSO_4$/ modified propylene microporous membrane	34.3 mF/cm^2, 9.95 mAh/cm^2) at 20 mV/s	84% after 10k cycles	9.34 µWh/cm^2 at 0.48 mW/cm^2, 6.61 µWh/cm^2 at 34 mW/cm^2	
Siloxane	Zn foil		6.86 mF/cm^2	94.3% over 16000 cycles	10.66 mJ/cm^2 (max.) and 4.50 mW/cm^2 (max.)	S3
Oxidized carbon nanotube	Zn foil	$ZnSO_4$–polyvynyl alcohal, 0–1.8 V	20 mF/cm^2 (53 F/g) at 10 mV/s	5000 cycles at 0.5 V/s	-	S4
Oxidized carbon nanotube	Zn foil	1 M $ZnSO_4$	15 mF/cm^2 at 10 mV/s	5000 cycles at 0.5 V/s	-	
Laser-mediated Explosive Synthesis and Transfer of graphene	Zn foil	2M $Zn(CH_3COO)_2$, 0.2-1.6 V	~21 µAh/cm^2 at 10 mV/s	73.8% for 10k cycles at 2 mA/cm^2	3.06* µWh/cm^2 at 0.074* mW/cm^2, 1.09* µWh/cm^2 at 4.95* mW/cm^2	S5
SiC/SiO_x	3D Zn nanoflakes	1.8 V, 6M $ZnCl_2$	124.7 mF/cm^2 at 10 mV/s	97.8% after 10k cycles	54.1 µWh/cm^2 at 1.08 mW/cm^2; 41.8 µWh/cm^2 at 16.7 mW/cm^2	S6
MoS_2 nanosheets/poly(3-thiophene methyl acetate) composite		2M $ZnSO_4$/Whatman GF/B separator.	28.8 mF/cm^2 at 0.028 mA/cm^2	>100% after 10k cycles at 0.028 mA/cm^2	-	S 7
Vertical Ti2CTx MXene@carbon fiber	Zn@Cu@CF devices	2M $ZnSO_4$/cellulose diaphragm, 0-1 V	380 mF/cm^2 at 5 mV/s.	90% after 10k cycles	52.77 µWh/cm^2 at 0.950 mW/cm^2	S 8
Zn_xMnO_2 nanowires	Activated carbon cloth	2M $ZnSO_4$ + 0.4M $MnSO_4$, 0-2 V	1446.6 mF/cm^2 at 1 mA/cm^2	83.1% after 5k at 15 A/g	-	S 9
Ti3C2Tx-MXene	Zn foil	$ZnSO_4$-polyacrylamide hydrogel, 0-1.2 V	318 µF/cm^2 at 5 mV/s	76% after 10k	0.0513 µWh/cm^2 at 1.103 µW/cm^2	S 10

Reference

(1) Henderson, J. D.; Buchanan, S. D. C.; Grey, L. H.; Biesinger, M. C. Zinc and Cadmium: XPS Chemical State Determination and Auger Peak Curve-Fitting Procedures. *Appl. Surf. Sci.* **2026**, *730*, 166284. https://doi.org/10.1016/j.apsusc.2026.166284.

(2) Pal, A.; Dey, T. K.; Singhal, A.; Bindal, R. C.; Tewari, P. K. Nano-ZnO Impregnated Inorganic–Polymer Hybrid Thinfilm Nanocomposite Nanofiltration Membranes: An Investigation of Variation in Structure, Morphology and Transport Properties. *RSC Adv.* **2015**, *5* (43), 34134–34151. https://doi.org/10.1039/C4RA14854A.

(3) Guo, Q.; Han, Y.; Chen, N.; Qu, L. Few-Layer Siloxene as an Electrode for Superior High-Rate Zinc Ion Hybrid Capacitors. *ACS Energy Lett.* **2021**, *6* (5), 1786–1794. https://doi.org/10.1021/acsenergylett.1c00285.

(4) Tian, Y.; Amal, R.; Wang, D.-W. An Aqueous Metal-Ion Capacitor with Oxidized Carbon Nanotubes and Metallic Zinc Electrodes. *Front. Energy Res.* **2016**, *4* (OCT). https://doi.org/10.3389/fenrg.2016.00034.

(5) Samartzis, N.; Bhorkar, K.; Athanasiou, M.; Sygellou, L.; Dracopoulos, V.; Ioannides, T.; Yannopoulos, S. N. Direct Laser-Assisted Fabrication of Turbostratic Graphene Electrodes: Comparing Symmetric and Zinc-Ion Hybrid Supercapacitors. *Carbon N. Y.* **2023**, *201*, 941–951. https://doi.org/10.1016/j.carbon.2022.09.076.

(6) Li, W.; Yang, Z.; Chen, S.; Jiang, L.; Wang, L.; Liu, Q.; Yang, W. Crystal Plane Engineering of Zn Nanosheet Arrays Toward Robust Hybrid Capacitive Energy Storage. *ENERGY Environ. Mater.* **2026**. https://doi.org/10.1002/eem2.70248.

(7) Gangopadhyay, B.; Upreti, B. B.; Ali, M. S.; Das, A.; Saroj, V. K.; Panda, S.; Mallik, A.; Dey, R. S.; Chattopadhyay, D. MoS2 Nanosheets Pure Phase and Mixed Phase Created through the Intercalation of 3-Polythiophene Methyl Acetate (3-PTMA) and Their Effects on Zinc Ion Storage. *J. Energy Storage* **2026**, *168*, 122645. https://doi.org/10.1016/j.est.2026.122645.

(8) Shi, B.; Chen, L.; Jen, T.-C.; Liu, X.; Li, L.; Chen, A.; Shen, G. Vertical Arrangement of Ti 2 CT x MXene Nanosheets on Carbon Fibers for High-Performance and Flexible Zn-Ion Supercapacitors. *ACS Appl. Nano Mater.* **2023**, *6* (1), 315–322. https://doi.org/10.1021/acsanm.2c04422.

(9) Chen, Q.; Jin, J.; Kou, Z.; Liao, C.; Liu, Z.; Zhou, L.; Wang, J.; Mai, L. Zn 2+ Pre-Intercalation Stabilizes the Tunnel Structure of MnO 2 Nanowires and Enables Zinc-Ion Hybrid Supercapacitor of Battery-Level Energy Density. *Small* **2020**, *16* (14). https://doi.org/10.1002/smll.202000091.

(10) Huang, L.; Lin, Y.; Zeng, W.; Xu, C.; Chen, Z.; Wang, Q.; Zhou, H.; Yu, Q.; Zhao, B.; Ruan, L.; Wang, S. Highly Transparent and Flexible Zn-Ti 3 C 2 T x MXene Hybrid Capacitors. *Langmuir* **2022**, *38* (19), 5968–5976. https://doi.org/10.1021/acs.langmuir.1c03370.
